# Supplementary material for: Host plant nutrition drives fitness outcomes in the cactus specialist Drosophila mettleri
Source: PLoS One. 2026 May 11;21(5):e0332982. doi: 10.1371/journal.pone.0332982 (PMC13160323; doi:10.1371/journal.pone.0332982)
Supplement: S1 Table — Table shows egg, pupa, and adult Drosophila mettleri results for the nutritional assay shown in Fig 1. (PDF) [file pone.0332982.s001.pdf]

S1 Table. Table of mean survival and development times (days). Table shows egg, pupa, and adult *D. mettleri* results for the nutritional assay showed in Fig 1.

	Cornmeal Control	Cornmeal Power	Cornmeal Exudate	Cornmeal Soil	Banana Control	Banana Powder	Banana Exudate	Banana Soil
Egg	1556	2391	2738	2436	2294	4388	2198	2857
Pupae	1176	1420	1771	1454	1752	2248	954	146
Adult	832	1169	1458	1259	1179	1999	766	122
Pupae survival	75.58	59.39	64.68	59.69	76.37	51.23	43.40	5.11
Adult survival	70.75	82.32	82.33	86.59	67.29	88.92	80.29	83.56
Egg to Pupa	13	12	12	13	12	12	11	14
Pupa to Adult	7	7	7	9	8	7	7	8
Egg to Adult	20	19	19	22	20	19	18	22
