# Supplementary material for: Host plant nutrition drives fitness outcomes in the cactus specialist Drosophila mettleri
Source: PLoS One. 2026 May 11;21(5):e0332982. doi: 10.1371/journal.pone.0332982 (PMC13160323; doi:10.1371/journal.pone.0332982)
Supplement: S2 Table — Type III ANOVA from generalized linear mixed models (GLMMs) testing the effects of cactus treatment and day on survival across developmental stages and diet types (cornmeal and banana). Significance codes: ***p < 0.001, **p < 0.01, *p < 0.05. (PDF) [file pone.0332982.s002.pdf]

S2 Table. Table of ANOVA results. Type III ANOVA from generalized linear mixed models (GLMMs) testing the effects of cactus treatment and day on survival across developmental stages and diet types (cornmeal and banana). Significance codes: ***p < 0.001, **p < 0.01, *p < 0.05.

			Chisq	Df	p value	Significance
Cornmeal	egg to adult	(Intercept)	17.7324	1	2.54E-05	***
		cactus	6.9501	3	0.07351	
		day	49.1951	4	5.32E-10	***
	egg to pupae	(Intercept)	165.919	1	< 2.2E-16	***
		cactus	127.732	3	< 2.2E-16	***
		day	71.197	4	1.27E-14	***
	pupae to adult	(Intercept)	55.713	1	8.39E-14	***
		cactus	79.331	3	< 2.2E-16	***
		day	15.252	4	4.21E-03	**
Banana	egg to adult	(Intercept)	6.4465	1	1.11E-02	*
		cactus	999.1411	3	< 2.2E-16	***
		day	80.7082	4	< 2.2E-16	***
	egg to pupae	(Intercept)	434.08	1	< 2.2E-16	***
		cactus	1767.43	3	< 2.2E-16	***
		day	143.34	4	< 2.2E-16	***
	pupae to adult	(Intercept)	3.7231	1	0.05367	
		cactus	230.4682	3	< 2.2E-16	***
		day	34.0699	4	7.21E-07	***
