# Supplementary material for: Host plant nutrition drives fitness outcomes in the cactus specialist Drosophila mettleri
Source: PLoS One. 2026 May 11;21(5):e0332982. doi: 10.1371/journal.pone.0332982 (PMC13160323; doi:10.1371/journal.pone.0332982)
Supplement: S3 Table — Estimated survival probabilities (%) across developmental stages (egg-to-adult, egg-to-pupae, pupae-to-adult) for each cactus treatment and diet type (cornmeal and banana). (PDF) [file pone.0332982.s003.pdf]

S3 Table. Table of post-hoc results. Estimated survival probabilities (%) across developmental stages (egg-to-adult, egg-to-pupae, pupae-to-adult) for each cactus treatment and diet type (cornmeal and banana).

			Survival (%)	95% CI	Group
Cornmeal	egg to adult	control	54.6	49.5 - 59.6	a
		exudate	52.4	47.6 - 57.1	a
		powder	50.5	45.6 - 55.4	a
		soil	51.1	46.3 - 56.0	a
	egg to pupae	control	75.9	72.2-79.2	c
		exudate	63.8	59.8 - 67.7	b
		powder	60.6	56.4 - 64.7	ab
		soil	58.8	54.6 - 62.9	a
	pupae to adult	control	73.2	68.2 - 77.7	a
		exudate	82.5	78.8 - 85.7	b
		powder	83.4	79.6 - 86.6	bc
		soil	86.7	83.5 - 89.4	c
Banana	egg to adult	control	50.48	46.5 - 54.4	d
		exudate	36.52	32.9 - 40.4	b
		powder	45.3	41.7 - 49.0	c
		soil	4.15	3.3 - 5.2	a
	egg to pupae	control	76.54	74.5 - 78.5	d
		exudate	44.51	42.0 - 47.1	b
		powder	52.39	50.4 - 54.4	c
		soil	4.63	3.9 - 5.9	a
	pupae to adult	control	67.2	60.0 - 73.7	a
		exudate	83.2	77.8 - 87.4	b
		powder	87.8	83.9 - 90.8	c
		soil	86.5	78.9 - 91.7	bc
