# Supplementary material for: Host plant nutrition drives fitness outcomes in the cactus specialist Drosophila mettleri
Source: PLoS One. 2026 May 11;21(5):e0332982. doi: 10.1371/journal.pone.0332982 (PMC13160323; doi:10.1371/journal.pone.0332982)
Supplement: S4 Table — Count indicates the number of colonies found. Total area refers to the total area of microbial coverage. Average size refers to the average size of colonies. %Area indicates the percentage colony cover compared to the total area. Mean refers to the average pixel value. (PDF) [file pone.0332982.s004.pdf]

S4 Table. Table of agar plate readings. Count indicates the number of colonies found. Total area refers to the total area of microbial coverage. Average size refers to the average size of colonies. %Area indicates the percentage colony cover compared to the total area. Mean refers to the average pixel value.

Slice	Number of Colonies	Total Area	Average Size	%Area	Mean
YPD control	1	92087	92087	100	255
YPD cactus powder	22	17363	42.246	14.81	255
YPD cactus exudate	1155	96834	83.839	51.665	255
YPD cactus soil	531	87878	165.495	60.33	255
BHI control	1	98617	98617	100	255
BHI powder	1	83272	83272	100	255
BHI cactus exudate	1937	82090	42.38	42.986	255
BHI cactus soil	1256	93741	74.635	45.985	255
LB control	1	98065	98065	100	255
LB powder	1	25643	20.78	9.92	255
LB cactus exudate	1706	82808	48.539	43.463	255
LB cactus soil	1273	92677	72.802	45.971	255
MH control	1	109064	109064	100	255
MH powder	1	85887	85887	100	255
MH cactus exudate	2042	75182	36.818	40.675	254.925
MH cactus soil	888	60871	68.548	44.682	255
