# Supplementary material for: Host plant nutrition drives fitness outcomes in the cactus specialist Drosophila mettleri
Source: PLoS One. 2026 May 11;21(5):e0332982. doi: 10.1371/journal.pone.0332982 (PMC13160323; doi:10.1371/journal.pone.0332982)
Supplement: Attachment — Submitted filename: Response to Reviewers.pdf [file pone.0332982.s007.pdf]

We thank the reviewer and editor for their thoughtful and constructive feedback. Below, we provide a point-by-point response. Reviewer comments are in black, followed by author responses in green. Line numbers refer to “Revised Manuscript (clean copy)”.

Journal Requirements:

1. If the reviewer comments include a recommendation to cite specific previously published works, please review and evaluate these publications to determine whether they are relevant and should be cited. There is no requirement to cite these works unless the editor has indicated otherwise.

No reviewer-suggested citations were provided.

2. Please review your reference list to ensure that it is complete and correct. If you have cited papers that have been retracted, please include the rationale for doing so in the manuscript text, or remove these references and replace them with relevant current references. Any changes to the reference list should be mentioned in the rebuttal letter that accompanies your revised manuscript. If you need to cite a retracted article, indicate the article’s retracted status in the References list and also include a citation and full reference for the retraction notice.

We have reviewed the reference list to ensure that it is complete and up to date. We confirm that none of the cited works have been retracted to our knowledge.

We have added several new references (Refs. 43-47) to support the revised statistical analyses, including sources for R, GLMM methodology, and the packages lme4, car, and emmeans. These additions are now cited in the Methods section lines (130, 133-134, 136-139).

Review Comments to the Author

Please use the space provided to explain your answers to the questions above. You may also include additional comments for the author, including concerns about dual publication, research ethics, or publication ethics. (Please upload your review as an attachment if it exceeds 20,000 characters)

Reviewer #1: I appreciate that the author’s attempted to address my previous comments, but I still have a some concerns that I think need to be further addressed.
The authors conducted the GLMM for egg to adult survival as suggested. However, they still treat this as secondary to the egg to pupae, and pupae to adult analyses. It seems to me

that it makes the most sense to start with egg-adult as the overall measure of fitness and then deconstruct it into stage specific analyses if they want to highlight how survival varies across developmental stage. Most importantly, the stage-specific analyses should also utilize GLMM instead of the Kruskall-Wallis test. A final point: the authors presented the results of the GLMM as a series of parameter estimates. I think they should consider summarizing the analysis with an anova table (e.g. the ‘car’ package can be used to produce an anova table of GLMM) and potential post hoc follow-up tests as this may be more easily digestible by readers. They may also consider analyzing the banana and cornmeal experiments separately.

We thank the reviewer for these constructive suggestions and have revised the manuscript accordingly in several ways.

We now clarify that all survival analyses (egg-to-pupae, pupae-to-adult, and cumulative egg-to-adult) were conducted using a generalized linear mixed-effects model (GLMM) with a binomial distribution, ensuring a consistent analytical framework across life stages. Cornmeal and banana responses were analyzed separately to assess diet related survival. To improve interpretability, we report results using Type III ANOVA tables (via the car package) along with estimated marginal means and post hoc contrasts (via emmeans), rather than focusing on individual parameter estimates (see Fig. 2 and Supporting Tables S2-S3).

We also appreciate the suggestion to emphasize egg-to-adult survival as the overall measure of fitness. In the revised manuscript, we have clarified the presentation to more explicitly link stage-specific results to cumulative egg-to-adult outcomes. While stage-specific survival is presented to highlight differences across developmental stages, these results are integrated within the same figure and Discussion section to emphasize their contribution to overall fitness.

2. I do not see a figure 4 although it is referenced in the text.

We thank the reviewer for catching this omission. Figure 4 has now been included in the revised manuscript materials and is properly referenced in the Results section.

3. Lines 258-260: I do not understand how egg to adult survival reflects the consequences of parental feeding behavior. Perhaps it is meant to be parental oviposition behavior?

We agree that egg-to-adult survival is more directly linked to parental oviposition behavior, as adults determine the developmental environment experienced by their offspring. We have revised the text accordingly to reflect this interpretation (lines 252-253).
